# Coumarin-Based Fluorescence Probe for Differentiated Detection of Biothiols and Its Bioimaging in Cells

**DOI:** 10.3390/bios13040447

**Published:** 2023-03-31

**Authors:** Wei Du, Xiu-Lin Gong, Yang Tian, Xi Zhu, Yu Peng, Ya-Wen Wang

**Affiliations:** 1 School of Life Science and Engineering, School of Chemistry, Southwest Jiaotong University, Chengdu 610031, China; 2 Department of Neurology, The Third People’s Hospital of Chengdu, The Affiliated Hospital of Southwest Jiaotong University, Chengdu 610031, China

**Keywords:** biothiols, fluorescence, coumarin derivative

## Abstract

In this work, a coumarin derivative, **SWJT-14**, was synthesized as a fluorescence probe to distinguish cysteine (Cys), homocysteine (Hcy) and glutathione (GSH) in aqueous solutions. The detection limit of Cys, Hcy and GSH for the probe was 0.02 μM, 0.42 μM and 0.92 μM, respectively, which was lower than biothiols in cells. The probe reacted with biothiols to generate different products with different conjugated structures. Additionally, it could distinguish Cys, Hcy and GSH using fluorescence and UV-Vis spectra. The detection mechanism was confirmed by MS. **SWJT-14** was successfully used in cellular experiments and detected both endogenous and exogenous biothiols.

## 1. Introduction

The biothiols such as Cysteine (Cys), homocysteine (Hcy) and glutathione (GSH) are associated with many physiopathologies in living organisms. Cys helps maintain redox homeostasis and regulates intracellular and intercellular signaling [1,2,3,4,5], Hcy is a direct independent diagnostic factor for many diseases [6,7,8,9,10] and GSH plays an important role in preventing or reducing the effects of harmful ROS in cardiovascular disease [11]. Biothiols are involved in a variety of biometabolic processes in human beings, such as oxidative stress, protein modification, signal transduction, cell apoptosis and cell differentiation. Their abnormal levels are related to many chronic and degenerative diseases [12,13,14,15,16,17].

A variety of methods have been developed for the detection of biothiols [18,19,20,21], most of which have the disadvantages of being inconvenient, time-consuming and non-specific. Fluorescence detection technology based on fluorescence probes can take images of analytes in situ and has the advantages of simple operation and low cost; thus, it developed rapidly. In order to target the identification of Cys, Hcy and GSH, the general approach is to exploit the nucleophilic nature of biothiols. Therefore, some organic reactions have been used to design chemodosimeters as fluorescent probes, such as the cyclization of aldehydes [22,23], Michael addition [24,25], conjugate addition cyclization with acrylates [26,27], aromatic nucleophilic substitution–rearrangement reactions [28], cleavage of sulphonamides and sulphonates [29,30], as well as metal complexation [31,32] and dithiol breakage reactions [33,34]. However, most of the reported probes can only identify one biothiol or do not discriminate between the three biothiols. Thus, chemodosimeters with multisite recognition have been developed as fluorescence probes to discriminate biothiols [35,36,37,38,39,40] (Table S1, Supplementary Materials). Although these probes were good differentiators for the three biothiols, differentiating them in the high water phase remained a great challenge.

**Scheme 1:**
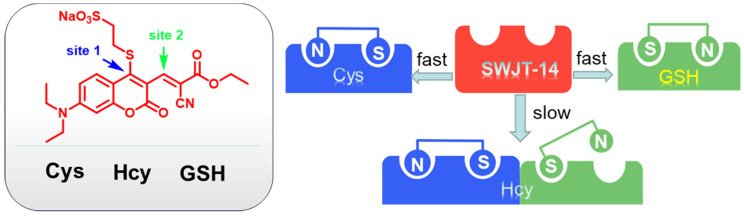
Response of **SWJT-14** to biothiols.

In connection with our continuing research on biothiols [41,42,43], herein we report a coumarin derivative as a fluorescence probe to distinguish three biothiols in an aqueous solution. Sodium 2-mercaptoethanesulfonate was introduced as a leaving group on coumarin, which can increase the aqueous solubility of the probe. It also synergistically provided multiple reaction sites with double bonds. The reaction of the probe with three biothiols could produce different products with different conjugated structures (Scheme 1), resulting in different UV-Vis changes and fluorescence changes at different excitation wavelengths for the purpose of the identification of the three biothiols. The reaction mechanism was confirmed by mass spectrometry and TD-DFT calculations. Furthermore, the probe could be used to image biothiols in HeLa cells.

## 2. Synthesis of Probe SWJT-14

**Scheme 2:**
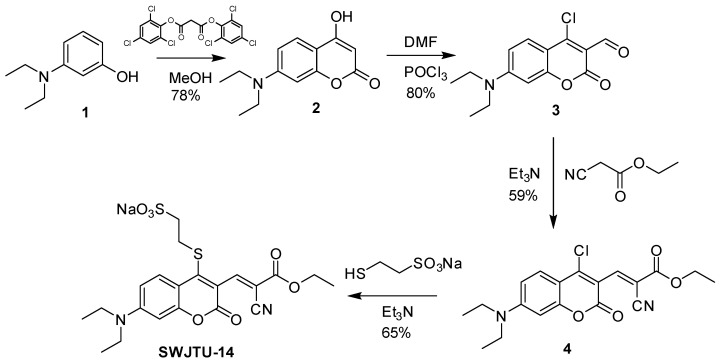
Syntheses of probe **SWJT-14**.

The synthetic route was shown in Scheme 2. Compounds **2**–**4** were obtained based on the published literature [36,44,45].

A solution of sodium 2-mercaptoethanesulfonate (52 mg, 0.317 mmol) in 1 mL water was added to a solution of **4** (100 mg, 0.267 mmol) in 4 mL acetonitrile and stirred for 5 min, then two drops of triethylamine were added to the mixture. After stirring at room temperature for 15 min, the solvents were removed by evaporation and the residue was purified by column chromatography (DCM:MeOH = 15:1) to obtain red solids (87 mg, yield: 65%). ^1^H NMR (D_2_O, 400 MHz) δ = 8.18 (s, 1H), 7.55 (d, *J* = 9.2 Hz, 1H), 6.53 (d, *J* = 8.4 Hz, 1H), 6.17 (brs, 1H), 4.38 (q, *J* = 6.4 Hz, 2H), 3.35 (m, 4H), 3.25–3.15 (m, 2H), 3.10–3.02 (m, 2H), 1.41 (t, *J* = 6.4 Hz, 3H), 1.13 (t, *J* = 7.2 Hz, 6H) ppm.^13^C NMR (DMSO-*d*_6_, 100 MHz) δ = 162.77, 158.23, 156.51, 155.91, 153.28, 149.80, 130.19, 115.00, 114.74, 111.01, 109.62, 106.23, 96.90, 62.76, 51.84, 44.97, 33.49, 14.49, 12.88 ppm. ESI-MS: *m*/*z* 479.06 [M − Na]^−^ (Figures S1–S3, Supplementary Materials).

## 3. Results

### 3.1. The Spectral Responses of **SWJT-14** for Biothiols in Aqueous Solution

The absorption and fluorescence spectra of **SWJT-14** in different solvents were used for the research (Table S2, Figures S4 and S5, Supplementary Materials). The absorption of **SWJT-14** in most solutions did not show recognition for biothiols. However, there was a clear distinction between the probe and the three biothiols in HEPES, Tris and PBS buffer solutions. In HEPES and Tris buffer solutions, the absorption peaks of the probe and GSH were almost the same. However, in the PBS buffer solution, **SWJT-14** exhibited a maximum absorption peak at 504 nm. Upon the addition of Cys, Hcy or GSH to the solution, the maximum absorption peak blue shifted to 380 nm, 460 nm and 490 nm, respectively. For fluorescence spectra, when the excitation wavelength was 380 nm, the fluorescence of the probe increased after the addition of three biothiols in HEPES, Tris and PBS buffer solutions. Then, the pH effect on the probe was measured. As shown in Figure S6 (Supplementary Materials), the **SWJT-14** and **SWJT-14** + biothiols system had good fluorescence in aqueous solutions and exhibited good stability from pH 2.0 to 11.0. Considering the application in biological systems, the PBS solution with pH 7.40 was selected for the subsequent experiment.

**Figure 1:**
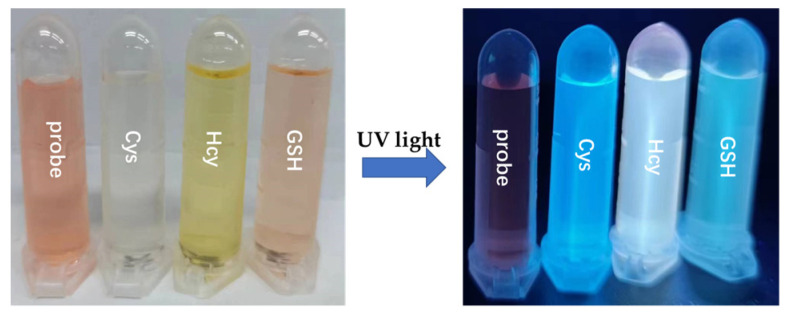
The photos of **SWJT-14** with Cys, Hcy and GSH in visible light and UV light at 365 nm.

In order to study the **SWJT-14** for three biothiols in detail, UV-Vis spectra (Figure S7, Supplementary Materials) and color changes (Figure 1) in PBS buffer solution were used. UV-Vis spectra of the probe + Cys system showed that the absorption peak at 504 nm decreased, accompanied by a new peak at 380 nm, enhanced within 20 min (Figure S7a, Supplementary Materials). For the probe + Hcy system, the absorption peak at 504 nm decreased, accompanied by new peaks at 460 nm and 375 nm, enhanced within 40 min (Figure S7b, Supplementary Materials). For the probe + GSH system, the absorption peak at 504 nm shifted to 490 within 20 min (Figure S7c, Supplementary Materials). As shown in Figure 1 (left), the color of the solution showed a distinct change from pink (**SWJT-14**) to colorless (**SWJT-14** + Cys), yellow (**SWJT-14**+ Hcy) and pale pink (**SWJT-14** + GSH). As shown in Figure 1 (right), the fluorescence color of the solution changed from colorless (**SWJT-14**) to blue (**SWJT-14** + Cys), green (**SWJT-14**+ Hcy) and pale green (**SWJT-14** + GSH). These results showed that **SWJT-14** could recognize the three biothiols in UV-Vis mode and by the naked eye.

**Figure 2:**
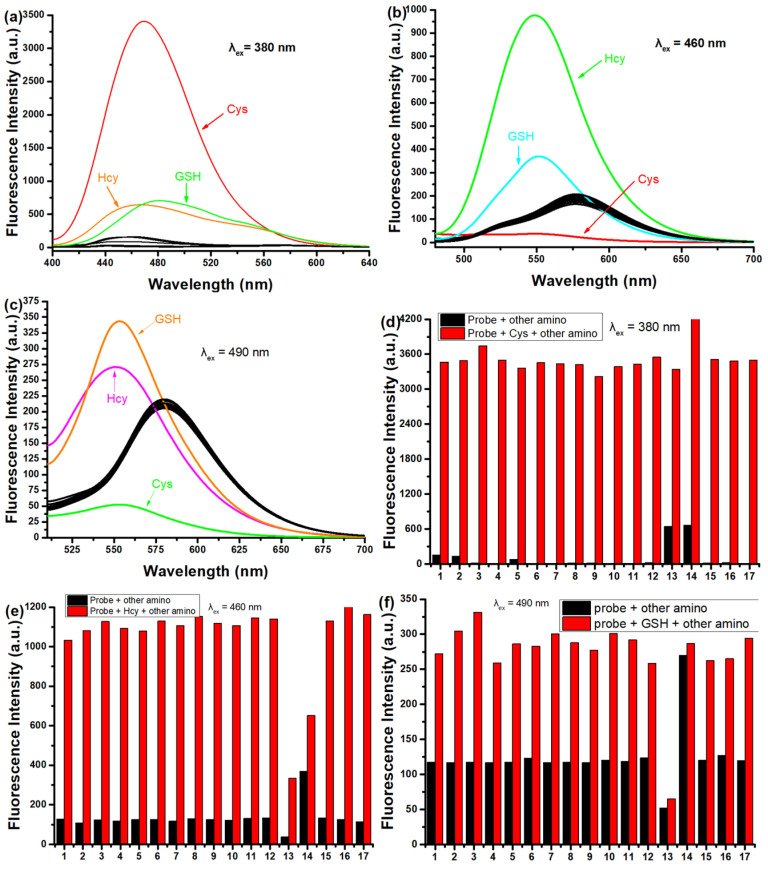
(**a**–**c**) Fluorescence responses and (**d**,**e**) competitive responses of **SWJT-14** (10.0 µM) with different analytes in PBS buffer solution (0.5% DMSO, pH 7.40). Analytes: Met, Leu, Pro, Thr, Try, Arg, Glu, Gly, Phe, Ser, Ala, H_2_S, Cys, Hcy, GSH, Lys, His and Et_3_N. (**a**,**d**) λ_em_ = 470 nm, λ_ex_ = 380 nm (**b**,**e**) λ_em_ = 550 nm, λ_ex_ = 460 nm and (**c**,**f**) λ_em_ = 553 nm, λ_ex_ = 490 nm. (**e**,**f**) The black bars represent the fluorescence responses after the addition of the probe and other analytes. The red bars represent the fluorescent responses to the subsequent addition of (**d**) Cys, (**e**) Hcy or (**f**) GSH to the above solution.

Then, the fluorescence of **SWJT-14** for the three biothiols was studied in detail. The emission peak of **SWJT-14** itself at different excitation wavelengths showed almost no difference, and the photostability of the probe was good (Figure S8, Supplementary Materials). **SWJT-14** showed weak fluorescence at 584 nm, the quantum yield Ф [46,47] was 0.32% (λ_ex_ = 490 nm), 0.26% (λ_ex_ = 460 nm) and 0.06% (λ_ex_ = 380 nm). As shown in Figure 2a, compared with other analytes, upon the addition of Cys to the solution, the fluorescence enhanced at 470 nm (λ_ex_ = 380 nm, Ф = 2.8%) and Hcy and GSH made weak emission peaks at 467 nm and 481 nm, respectively. Notably, when the excitation wavelength was changed to 460 nm, the **SWJT-14** + Hcy system had a distinct emission wavelength at 550 nm (Ф = 2.3%) (Figure 2b). When the excitation wavelength was 490 nm, GSH exhibited a maximum emission peak at 553 nm (Ф = 1.67%) (Figure 2c). As shown in Figure 2d, the presence of other analytes had no effect on the recognition of Cys by **SWJT-14**. Due to different reaction rates, there was, to a certain extent, an effect on the recognition of Hcy or GSH by **SWJT-14** (Figure 2e,f). These results showed that **SWJT-14** could detect the three biothiols in fluorescence mode, and combined with UV and naked-eye recognition, the three biothiols could be distinguished.

To further investigate the change in fluorescence intensity with increasing concentrations of biothiols, titration experiments were made. As shown in Figure S9 (Supplementary Materials), with the addition of biothiols to the solution, the fluorescence intensity of **SWJT-14** was enhanced 311-fold for Cys at 470 nm (λ_ex_ = 380 nm), 13.7-fold for Hcy at 550 nm (λ_ex_ = 460 nm) and 2-fold for GSH at 553 nm (λ_ex_ = 490 nm). As shown in Figure S10 (Supplementary Materials), **SWJT-14** had a good linear relationship with biothiols at low concentrations, thus the detection limits were calculated to be 0.0287 μM for Cys, 0.422 μM for Hcy and 0.924 μM for GSH [43]. The kinetic studies of **SWJT-14** with the three biothiols were performed at 37 °C using the corresponding excitation wavelengths. As shown in Figure S11 (Supplementary Materials), the reaction process conformed to the pseudo first-order kinetic equation. The slope of the linear fit was the rate constant, which was calculated to be 10.98 min^−1^ for Cys, 0.326 min^−1^ for Hcy, and 0.442 min^−1^ for GSH [48].

### 3.2. Proposed Mechanism

**Figure 3:**
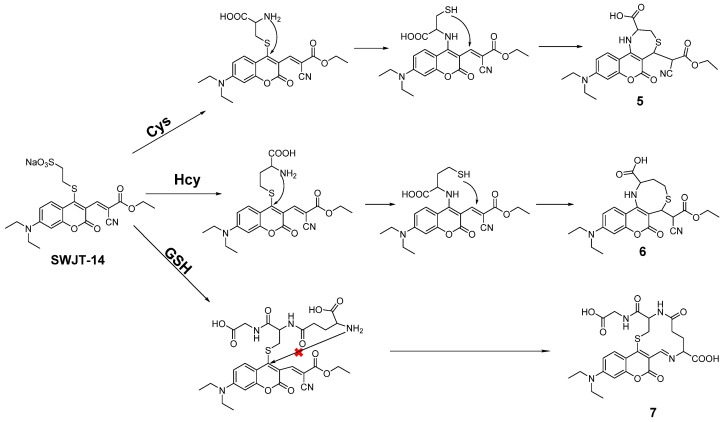
Proposed mechanism of **SWJT-14** to the biothiols.

According to the proposed mechanism [40,45], **SWJT-14** would react with biothiols to obtain the thiol derivatives, which were formed by replacing the mercaptosulfonic acid from the probe with sulfhydryl groups of biothiols, which quickly underwent intramolecular rearrangement to form a Schiff base (Figure 3). The exposed sulfhydryl groups further underwent a Michael addition reaction and formed structurally similar polycyclic products with Cys and Hcy. The difference in reaction rate was attributed to different potential space resistances. For GSH, the product underwent thiol coumarin and formed Schiff bases due to the large size and strong spatial site resistance of GSH. As shown in Figure S12 (Supplementary Materials), the peak of *m*/*z* 458.08 in MS was confirmed as a heptacyclic coumarin derivative was obtained after the reaction of **SWJT-14** with Cys. The peaks of *m*/*z* 472.13 and *m*/*z* 533.26 were the corresponding products of Hcy and GSH with **SWJT-14**, respectively (Figures S13 and S14, Supplementary Materials). These results indicated that different products were obtained, which was the origin of different spectral changes. The precise mechanism is still under study.

**Figure 4:**
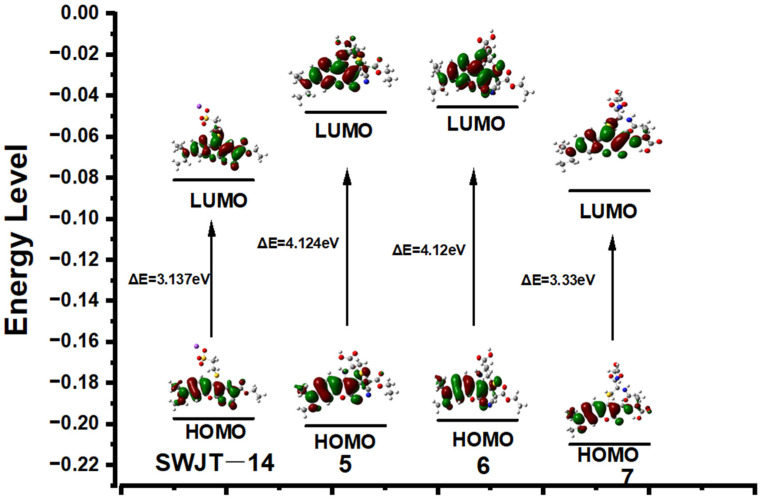
Energy levels and optimized structure of **SWJT-14**, **5**, **6** and **7**.

To investigate the relationship between spectra changes and structure, Gaussian calculations were performed. The optimized structures used the B3LYP/6-31G (d, p) basis set [49]. As shown in Figure 4, the electron density of LUMO was mainly distributed over coumarin and the adjacent ethyl cyanoacetate group. When the probe reacted with Cys or Hcy to obtain **5** or **6**, the decrease in electron density of the coumarin fraction indicated that the conjugated system was reduced and therefore higher energy excitation wavelengths were required. Compared with **5** or **6**, the electron density of **7** was similar to that of the probe, and therefore required a similar excitation wavelength to the probe. For **5**, **6** and **7**, the larger energy gap indicated a blue shift in absorption wavelength, which was consistent with the observed experimental results.

### 3.3. Bioimaging in Living Cells

**Figure 5:**
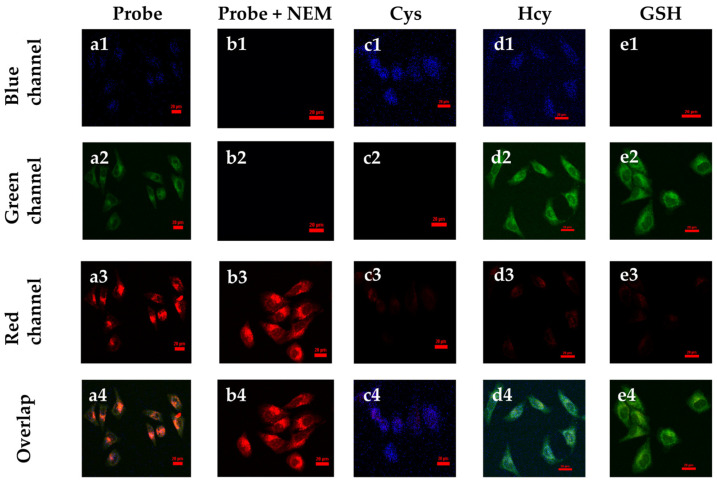
Confocal fluorescence images in HeLa cells. (**a1**–**a4**) Cells incubated with **SWJT-14** (20.0 μM) for 40 min. (**b1**–**b4**) Cells were incubated with NEM (200.0 μM) for 30 min, followed by the addition of **SWJT-14** and continued incubation for 40 min. (**c**–**e**) Cells were incubated with NEM for 30 min, followed by the addition of biothiol for 30 min and then the probe was added for 40 min. (**c1**–**c4**) Cys (200.0 μM), (**d1**–**d4**) Hcy (200.0 μM), (**e1**–**e4**) GSH (200.0 μM). Scale bar: 20.0 μm. λ_ex_ = 405 nm, λ_em_ = 425–475 nm for blue channel; λ_ex_ = 488 nm, λ_em_ = 500–550 nm for green channel; λ_ex_ = 561 nm, λ_em_ = 570–620 nm for red channel.

To further investigate the imaging of the SWJT-14 inside living cells, HeLa cells were selected for the experiments. As shown in Figure 5(a1–a4), faint fluorescence in the blue, green and red channels was observed after 40 min of co-incubation of the SWJT-14 with HeLa cells. After adding 200.0 μM of NEM (N-Ethylmaleimide, thiol blocking reagent) to block endogenous biothiols, the fluorescence of SWJT-14 itself in the red channel was shown (Figure 5(b1–b4)). When Cys was added to the cells that were shielded from endogenous biothiols, and incubated for 30 min, followed by incubation with SWJT-14 for 40 min, weak fluorescence in the blue channel and almost no fluorescence in the red or green channels was observed (Figure 5(c1–c4)). Hcy (Figure 5(d1–d4)) or GSH (Figure 5(e1–e4)) were added in the same way and showed fluorescence in the blue and green or green channels, while there was almost no fluorescence in the red channel. These results indicated that SWJT-14 could detect exogenous biothiols in living cells, which was consistent with the fluorescence detection results.

## 4. Conclusions

A coumarin derivative as a fluorescent probe was designed and synthesized. It can react with biothiols in aqueous solutions to generate products with different conjugated structures, resulting in different emission peaks at different excitation wavelengths, which achieved the purpose of differential detection of Cys, Hcy and GSH. The fluorescence imaging of **SWJT-14** had also been demonstrated in living cells.

## Data Availability

Not applicable.

## Supplementary Materials

The following supporting information can be downloaded at: https://www.mdpi.com/article/10.3390/bios13040447/s1, Copies of ^1^H, ^13^C NMR, ESI-MS spectra, spectral data and other materials. Table S1: Summary of representative probes for distinguishing of biothiols; Figure S1: 1H NMR spectrum (400 MHz, D2O) of SWJT-14; Figure S2: 13C NMR spectrum (100 MHz, DMSO-d6) of SWJT-14; Figure S3: LC-MS spectrum of probe SWJT-14; Table S2: Photophysical properties of SWJT-14; Figure S4: Absorption spectra of SWJT-14 (10.0 μM) with biothiols in different solvents; Figure S5: Fluorescence of SWJT-14 (10.0 μM) with biothiols in different solvents (λex = 380 nm); Figure S6: Fluorescence intensity changes before and after the addition of Cys, Hcy, GSH (200.0 μM) to the solution of SWJT-14 (10.0 μM, λex = 380 nm for Cys, λex = 460 nm for Hcy, λex = 380 nm for GSH) at different pH value; Figure S7: UV-vis absorption spectra of the time profiles. (a) SWJT-14 (10.0 μM) with Cys, (b) SWJT-14 with Hcy, (c) SWJT-14 with GSH in PBS buffer (0.5% DMSO, pH = 7.4); Figure S8: Emission peaks of the probe under different excitations and photostability of the probe (λex = 490 nm, λem = 584 nm); Figure S9: Titration curves of SWJT-14 (10.0 μM) with (a) Cys, λex = 380 nm; (b) Hcy, λex = 460 nm; (c) GSH, λex = 490 nm; Figure S10: The detection limit of probe SWJT-14 (10.0 μM) to Cys (λex = 380 nm), Hcy (λex = 460 nm), GSH (λex = 380 nm); Figure S11: (a–c) Kinetic fitting of SWJT-14 (10.0 μM) to Cys, Hcy and GSH (200.0 μM); Figure S12: LC-MS of SWJT-14 + Cys; Figure S13: LC-MS of SWJT-14 + Hcy; Figure S14: LC-MS of SWJT-14 + GSH; Figure S15: The viability of HeLa cells was determined by cck8 assay after incubation with different concentrations of SWJT-14 for 24 h; Figure S16: Optimized structure of SWJT-14; Figure S17: Optimized structure of 5; Figure S18: Optimized structure of 6; Figure S19: Optimized structure of 7.
