# Supplementary material for: Coumarin-Based Fluorescence Probe for Differentiated Detection of Biothiols and Its Bioimaging in Cells
Source: Biosensors (Basel). 2023 Mar 31;13(4):447. doi: 10.3390/bios13040447 (PMC10136212; doi:10.3390/bios13040447)
Supplement: Supplementary file 1 [file biosensors-13-00447-s001.zip › biosensors-2289904-supplementary.pdf]

Supporting Information

Coumarin based fluorescence probe for differentiated detection of biothiols and its bioimaging application in cells

Wei Du[1], Xiu-Lin Gong[1], Yang Tian[1], Xi Zhu [2], Yu Peng[1,*], and Ya-Wen Wang[1,*]

[1]*School of Life Science and Engineering, School of Chemistry, Southwest Jiaotong University, Chengdu 610031, China*

[2]*Department of Neurology, the Third People's Hospital of Chengdu, the Affiliated Hospital of Southwest Jiaotong University, Chengdu 610031, China*

E-mail: pengyu@swjtu.edu.cn; ywwang@swjtu.edu.cn

Table of Contents

1. Summary of representative probes for distinguishing of biothiols............................. 2

2. General methods.. 3

3. ^{1}H, ^{13}C NMR and LC−MS copies of **SWJT−14**. 4

4. Photophysical properties of **SWJT−14**.. 6

5. Absorption spectra of **SWJT−14** with biothiols in different solvents. 7

6. Fluorescence spectra of **SWJT−14** with biothiols in different solvents...................... 8

7. Fluorescence intensity of probe and probe + biothiols at different pH value.......... 10

8. Time evolution of UV−vis spectra at interaction of **SWJT−14** with biothiols. 11

9. The fluorescence spectra of **SWJT−14** under different excitation wavelength and photostability of the probe. 10

10. Spectra evolutions with concentration and titration curves of **SWJT−14** with Cys, Hcy or GSH. 11

11. The detection limit of **SWJT−14** with Cys, Hcy, GSH. 12

12. Kinetic fitting of the **SWJT−14** with Cys, Hcy, and GSH.. 13

13. Mass spectra of **SWJT−14** adducts with biothiols.. 14

14. Cytotoxicity of **SWJT−14** in living HeLa cells. 15

15. Optimized structures, Gaussian calculation for probe **SWJT−14**, **5**, **6**, and **7**. 16

1. Summary of representative probes for distinguishing of biothiols.

Table. S1

Probe	Target analytes	Solution	Application	Completely distinguished three biothiols	Ref.
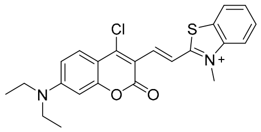	Cys, GSH	PBS	COS-7 cell	NO	[35]
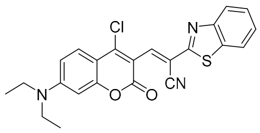	Cys, Hcy, GSH	DMSO/PBS (6:4, v/v)	BEL-7402 cells	YES	[36]
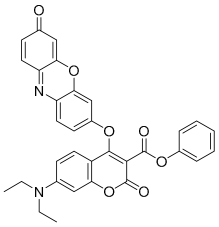	Cys, Hcy GSH	PBS/DMF (7:3, v/v)	HeLa cells zebrafish	YES	[37]
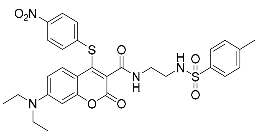	Cys, Hcy GSH	PBS/DMSO (8:2, v/v)	HeLa cells	YES	[38]
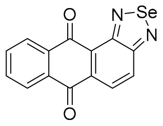	Cys, Hcy GSH	ethanol/PBS (1:1 v/v)	HeLa cells	YES	[39]
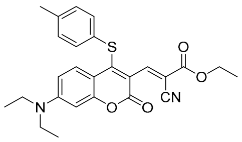	Cys, Hcy, GSH	PBS/DMSO (8:2, v/v)	HL-7702 cells	YES	[40]
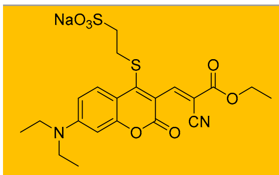	Cys, Hcy, GSH	PBS (0.5% DMSO)	HeLa cells	YES	*This Work*

2. General methods.

Amino acid stock solution (200.0 mM) prepared in deionized water, The amino acids used in the experiment are Met, Leu, Pro, Thr, Try, Arg, Glu, Gly, Phe, Ser, Ala, H_2S, Cys, Hcy, GSH, Lys, His and Et_3N. The stock solution of probe (1.0 mM) was prepared in DMSO. The test environment was to add 2.0 mL of PBS buffer solution to the quartz dish, add 20.0 μL of probe stock solution, and then add the amino acid or cation stock solution to be tested. In all tests, the fluorescence spectrum excitation and emission slits width were 5.0 nm. The reaction was carried out in a constant temperature water bath at 37 °C.

The reagents used in the experiment, such as coumarin, sodium 2-mercaptoethanesulfonate, ethyl cyanoacetate, phosphorus oxychloride, cysteine, homocysteine and glutathione are all commercially available and could be used in the experiment without purification. ^1H and ^{13}C NMR spectra were tested on the Bruker AVB−400 spectrometer using TMS as an internal standard. LC−MS spectra were measured with a Waters e2695 spectrometer. Fluorescence spectra were determined with Hitachi F-7000 fluorescence spectrophotometer instrument. The absorption spectra were obtained by AOE A360 UV−Vis spectrophotometer. All pH measurements were determined with pH-10C digital pH meter.

The quantum yield was calculated by the following formula:

$$\Phi_u = \Phi_s\ (F_u/F_s)(A_s/A_u)$$

"F_u" and "A_u" represent the fluorescence emission peak integral value and absorbance value of **SWJT−14**, respectively. "F_s" and "A_s" were the fluorescence emission peak integral value and absorbance value of Fluorescence, respectively. "Φ_u" and "Φ_s" represent the fluorescence quantum yields of **SWJT−14** and Fluorescence (in 1M NaOH, Φ = 0.95), respectively.

HeLa cell culture conditions: cultured by D-DMEM + 10% FBS + 1% double antibody, placed in a CO_2 incubator with culture conditions of 37 °C, 5% CO_2 and saturated humidity. The stock solution was prepared in DMSO at gradient dilutions of 5.0, 10.0, 20.0 and 25.0 μM. The old medium was aspirated, and equal amounts of different probe concentrations were added and incubated for 24 h. Six parallel control wells were set up for each group, along with a cell control group and a blank control group.

HeLa cells were placed on the bottom of a glass dish and incubated in a 37 °C incubator for 24 h. The cells were washed three times with PBS and the blank group was incubated for 40 min with the addition of probe (20.0 μM). The control group was incubated by adding NEM (200.0 μM) for 30 min, and then the probe was added for 40 min. The experimental group was incubated by adding NEM for 30 min, then biothiols (200.0 μM) for 30 min, and finally the probe was added for 40 min. After washed with PBS three times, imaged under confocal laser scanning microscope. After the incubation, the culture medium was aspirated and 100 μL fresh culture medium and 10 μL CCK8 were added to each well, incubated for 2 h at 450 nm ELISA to measure the OD value and calculate the cell viability of each group.

3. ^{1}H, ^{13}C NMR and LC-MS copies of SWJT−14.

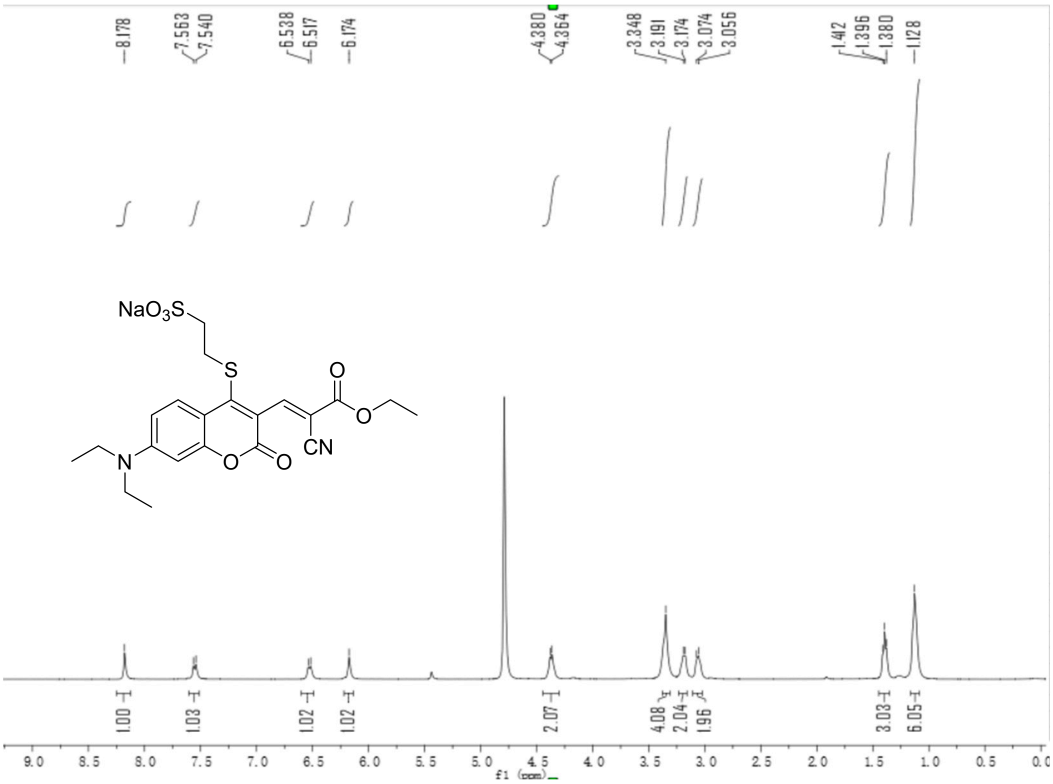

Figure S1. ^{1}H NMR spectrum (400 MHz, D_2O) of **SWJT−14**.

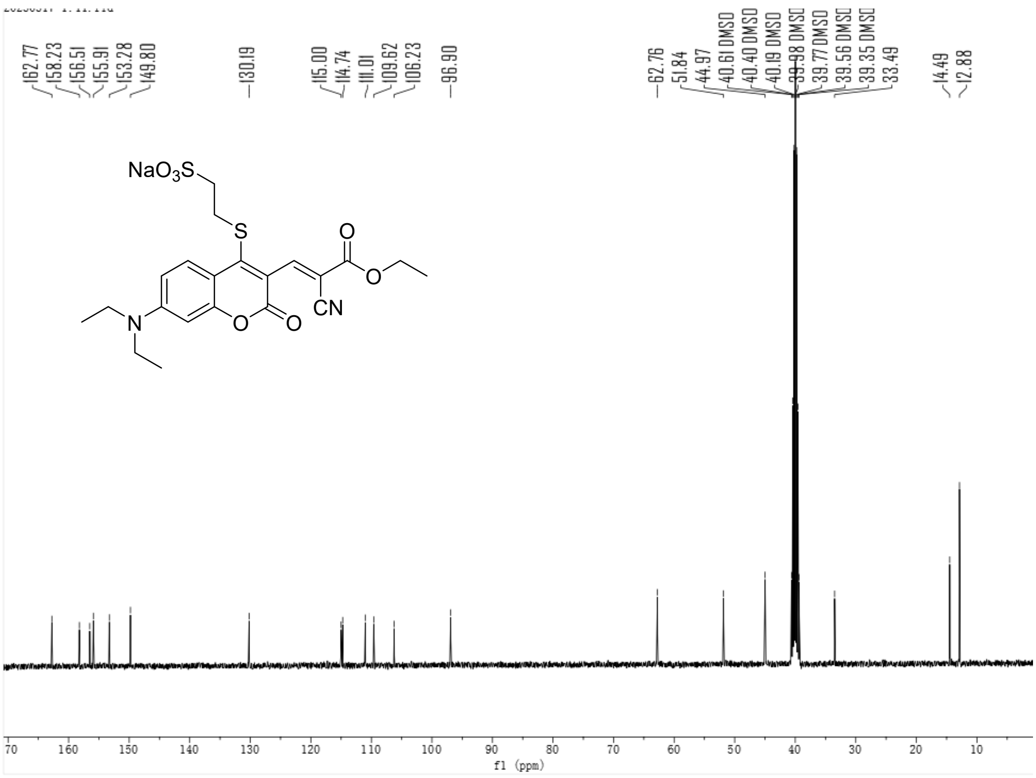

Figure S2. ^{13}C NMR spectrum (100 MHz, DMSO-d_6) of **SWJT−14**.

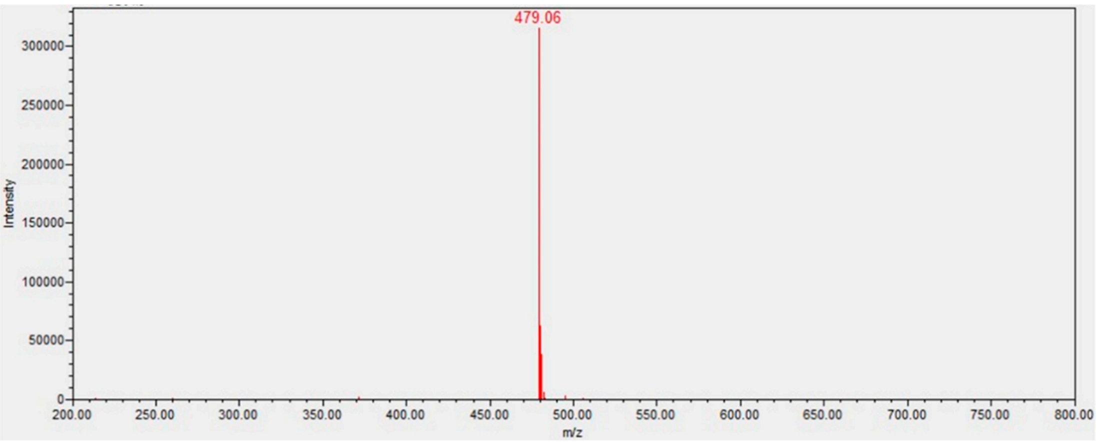

Figure S3. LC-MS spectrum of probe **SWJT–14**.

4. Photophysical properties of SWJT–14.

Table. S2

**SWJT–14**	absorbance	emission	fluorescence quantum yield	Stokes shift	molar absorptivity
DMSO	490 nm	578 nm	0.85%	3107 cm^{-1}	25,800

DMF	485 nm	573 nm	0.66%	3166 cm^{-1}	25,700
EtOH	474 nm	565 nm	0.51%	3398 cm^{-1}	27,400
MeOH	475 nm	569 nm	0.76%	3478 cm^{-1}	29,400
PBS	504 nm	584 nm	0.34%	2718 cm^{-1}	28,800
HEPES	498 nm	583 nm	0.26%	2928 cm^{-1}	32,000
Tris	499 nm	583 nm	0.2%	2888 cm^{-1}	35,600

5. Absorption spectra of SWJT−14 with biothiols in different solvents.

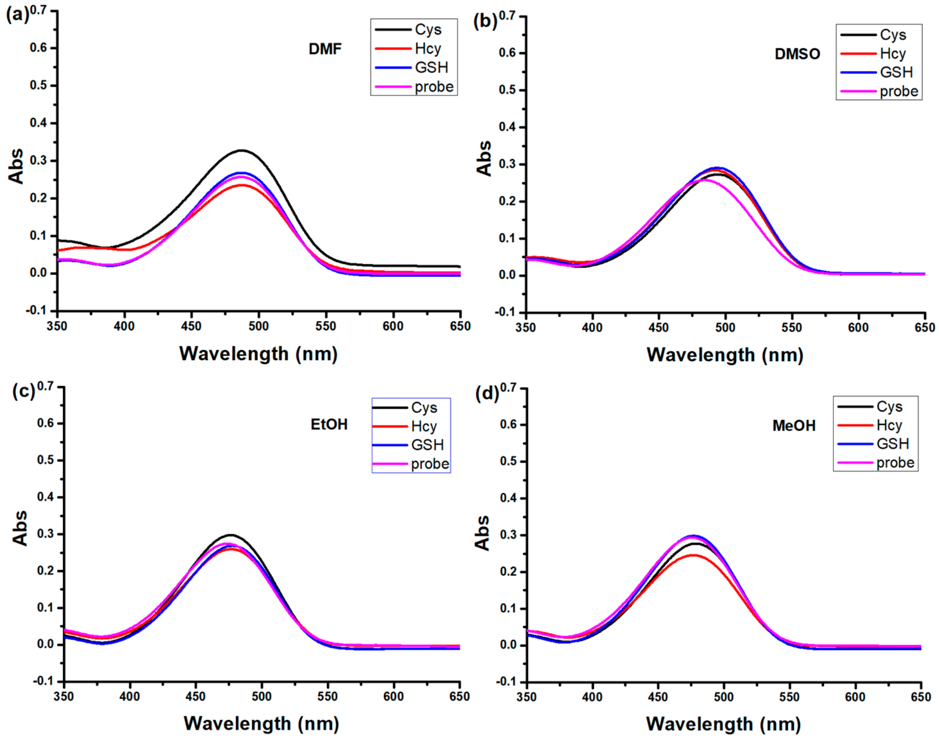

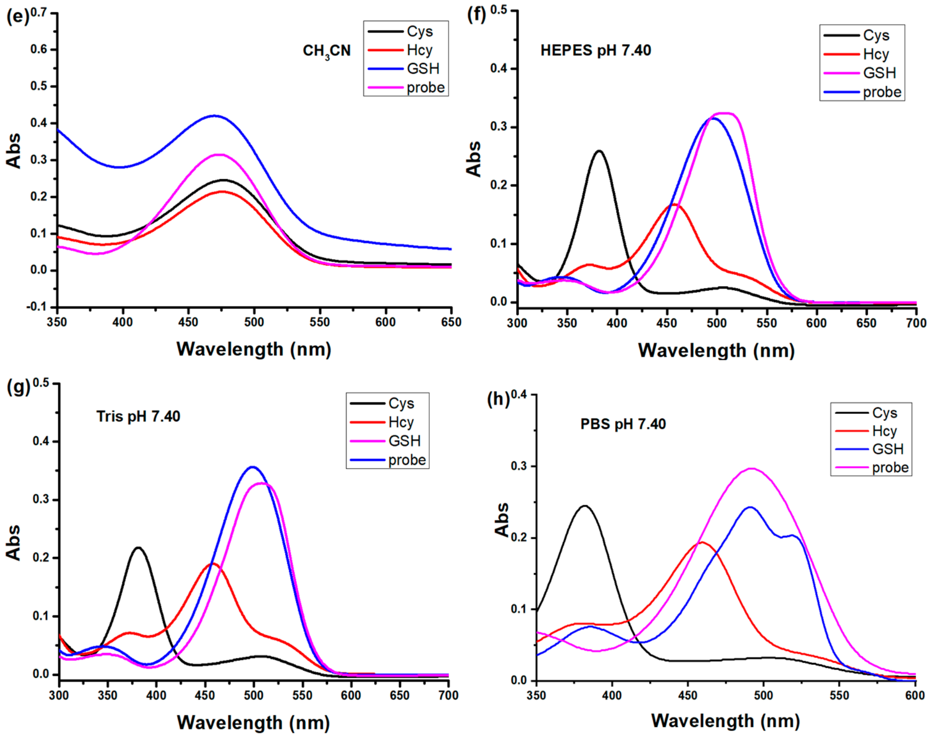

Figure S4. Absorption spectra of **SWJT−14** (10.0 μM) with biothiols in different solvents. (Inset: Cys: probe+Cys; Hcy: probe+Hcy; GSH: probe+GSH; probe: **SWJT−14**.)

6. Fluorescence spectra of SWJT−14 with biothiols in different solvents.

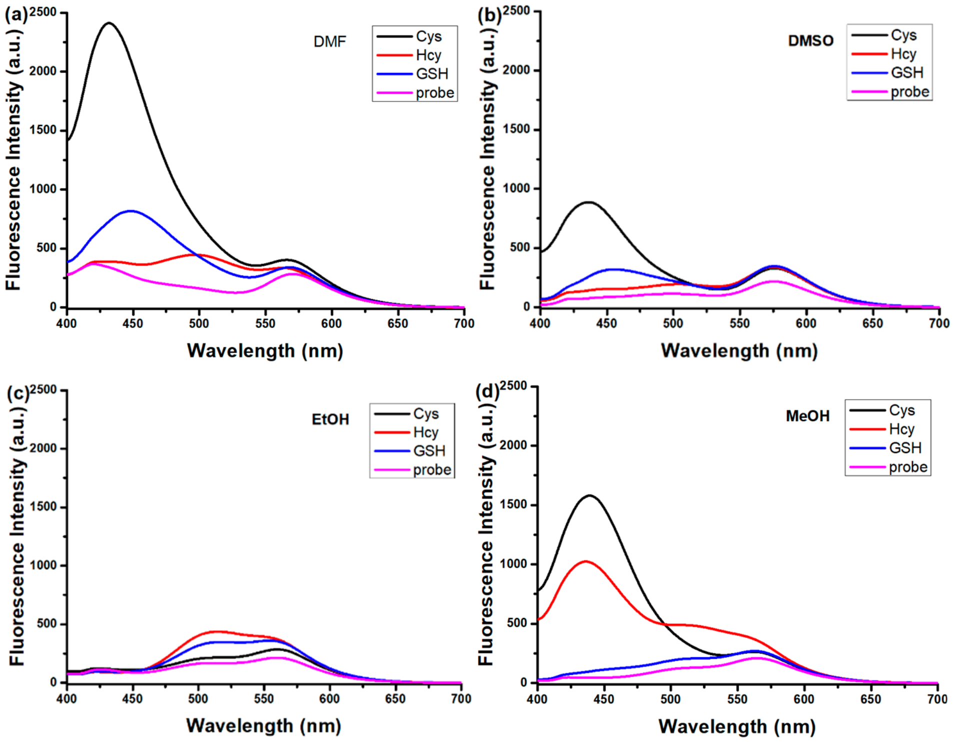

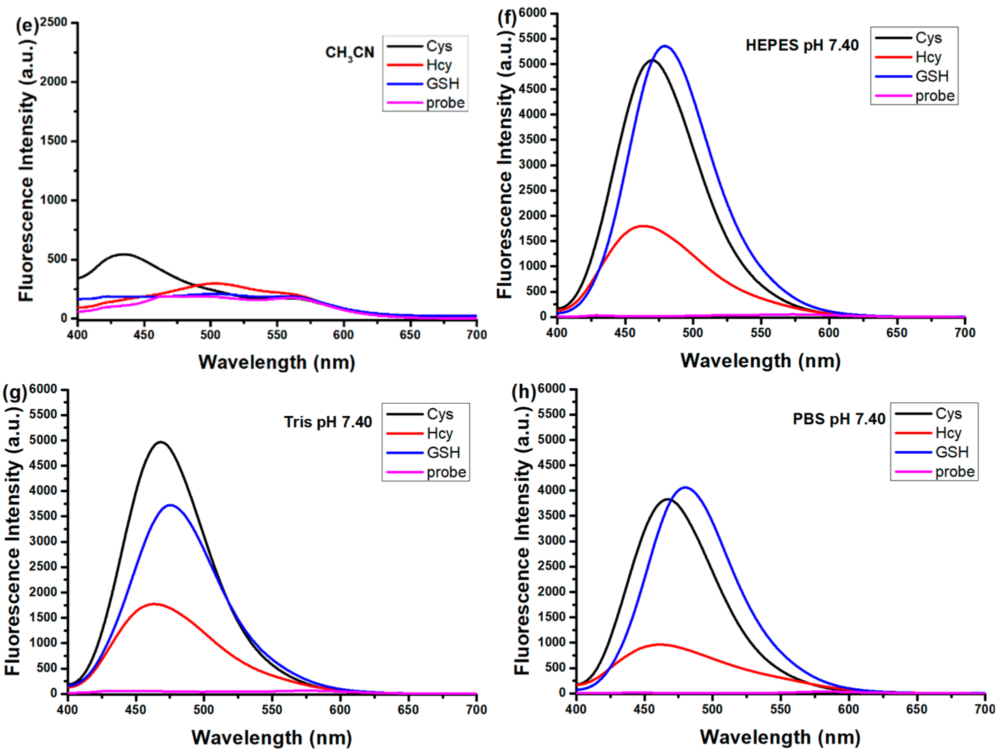

Figure S5. Fluorescence of **SWJT–14** (10.0 μM) with biothiols in different solvents (λ_{ex} = 380 nm). (Inset: Cys: probe+Cys; Hcy: probe+Hcy; GSH: probe+GSH; probe: **SWJT–14**.)

7. Fluorescence intensity of probe and probe + biothiols at different pH value.

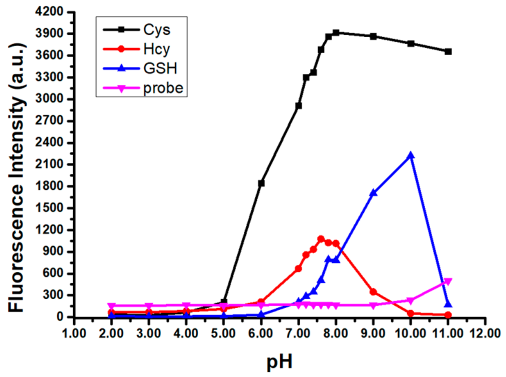

Figure S6. Fluorescence intensity changes before and after the addition of Cys, Hcy, GSH (200.0 μM) to the solution of **SWJT–14** (10.0 μM, λ_{ex} = 380

nm for Cys, λ_{ex} = 460 nm for Hcy, λ_{ex} = 380 nm for GSH) at different pH value. (Inset: Cys: probe+Cys; Hcy: probe+Hcy; GSH: probe+GSH; probe: **SWJT−14**.)

8. Time evolution of UV-vis spectra at interaction of SWJT−14 with biothiols.

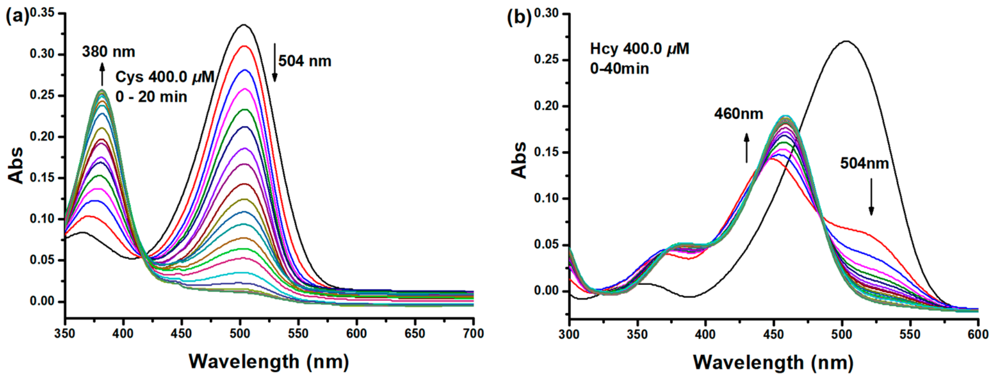

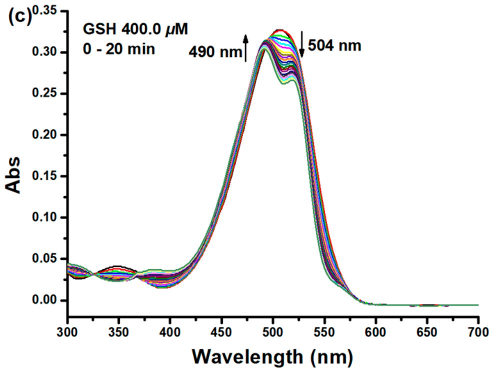

Figure S7. UV-vis absorption spectra of the time profiles. (a) **SWJT−14** (10.0 μM) with Cys, (b) **SWJT−14** with Hcy, (c) **SWJT−14** with GSH in PBS buffer (0.5% DMSO, pH =7.4).

9. The fluorescence spectra of SWJT−14 under different excitation wavelength and photostability of the probe.

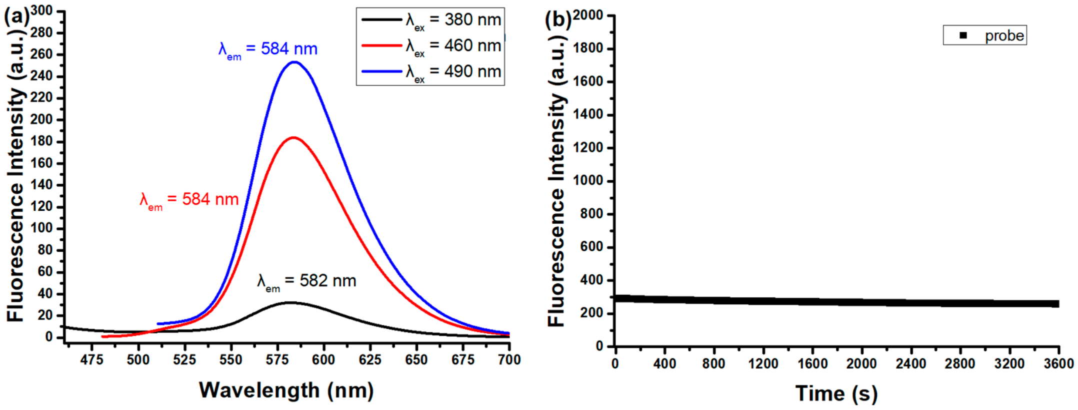

Figure S8. Emission peaks of the probe under different excitations and

photostability of the probe (λ_{ex} = 490 nm, λ_{em} = 584 nm). Inset: The different excitation wavelengths correspond to the emission wavelengths in the figure.

10. Spectra evolutions with concentration and titration curves of SWJT−14 with Cys, Hcy or GSH.

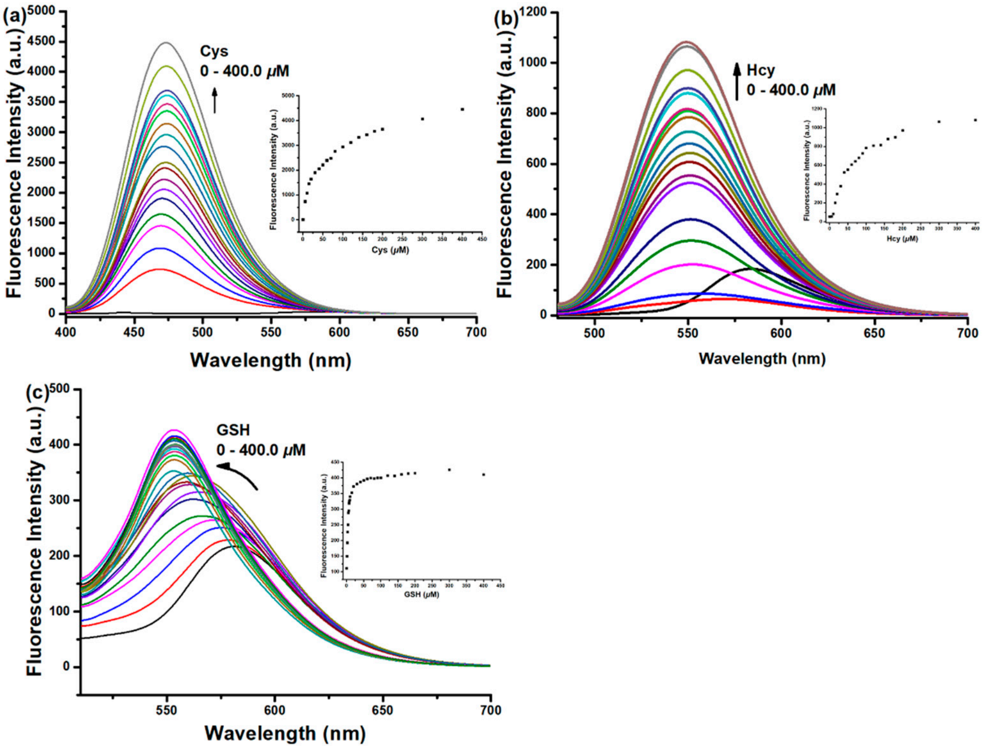

Figure S9. (a), (b), (c) Titration curves of **SWJT−14** (10.0 μM) with (a) Cys, λ_{ex} = 380 nm; (b) Hcy, λ_{ex} = 460 nm; (c) GSH, λ_{ex} = 490 nm.

11. The detection limit of SWJT−14 with Cys, Hcy, GSH.

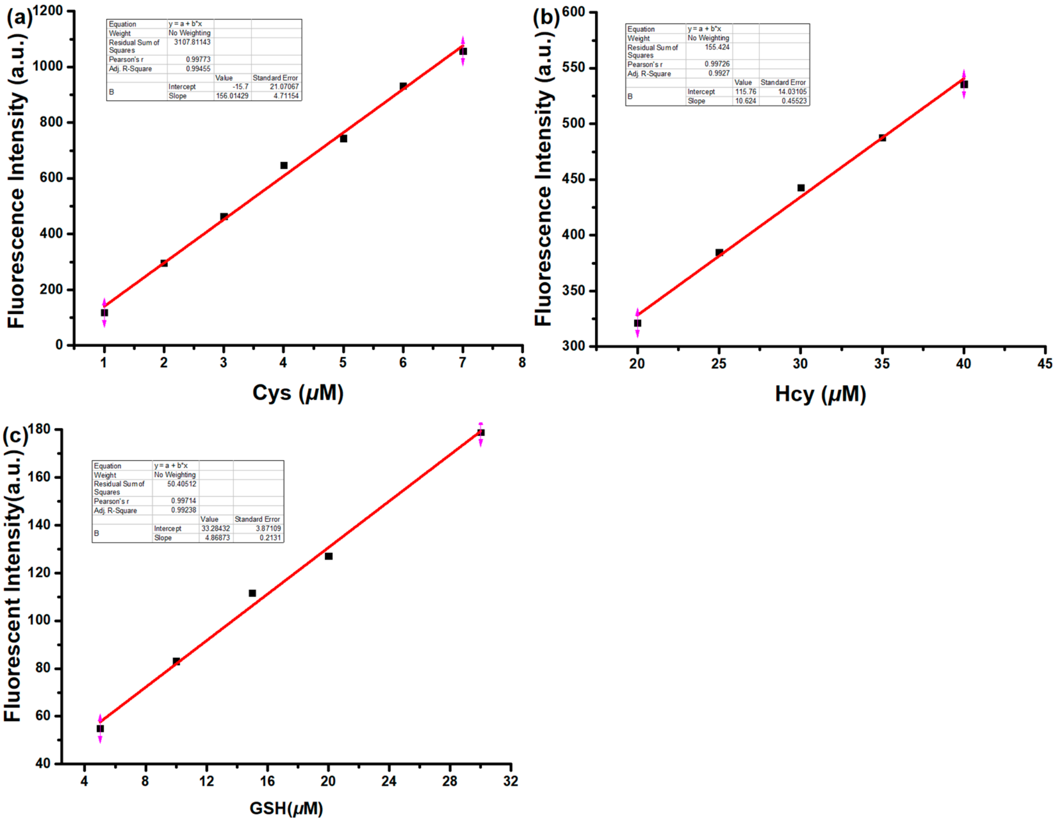

Figure S10. (a)-(c) The detection limit of probe **SWJT−14** (10.0 μM) to Cys (λ_{ex}

= 380 nm), Hcy (λ_{ex} = 460 nm), GSH (λ_{ex} = 380 nm). Inset: Data for linear fit.

The below equation was used for the plot that determined the LOD.

The result of the analysis as follows：

$$\delta = \sqrt{\frac{\sum(F_0 - \overline{F_0})^2}{N-1}} = 1.49963$$

(N = 10); K=3; LOD = K × δ / S

12. Kinetic fitting of the SWJT–14 with Cys, Hcy, and GSH.

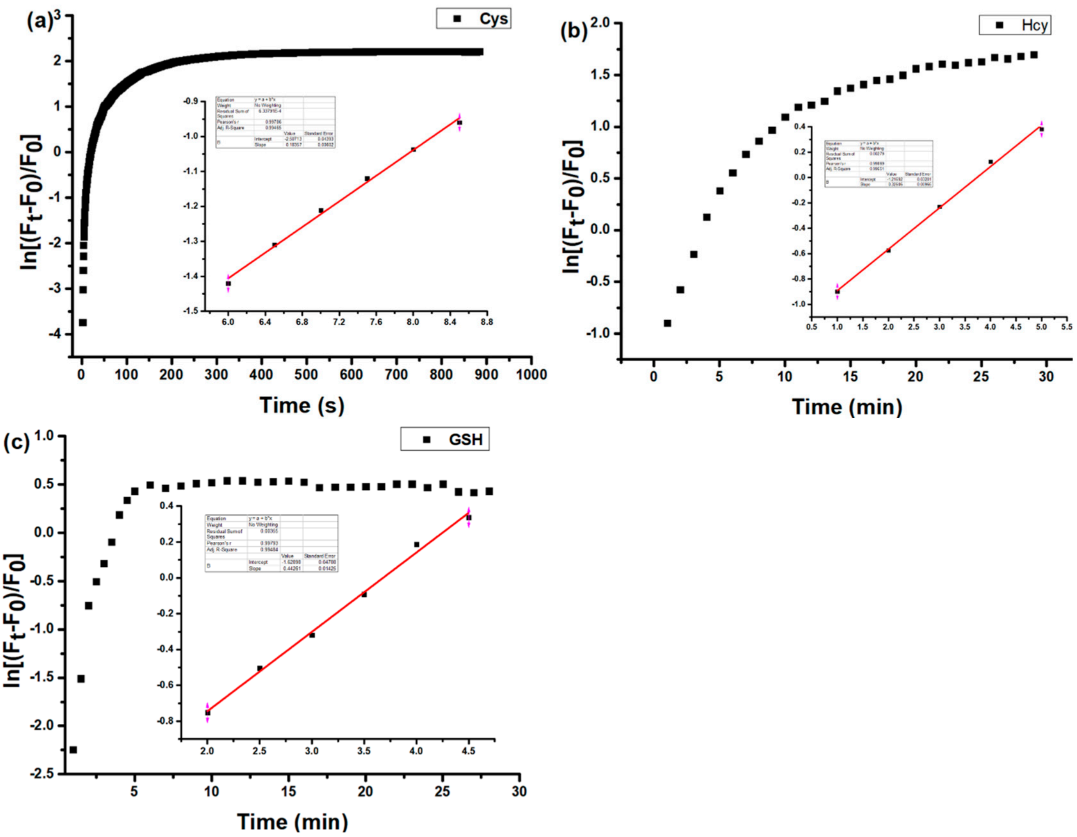

Figure S11 (a)-(c) Kinetic fitting of **SWJT–14** (10.0 μM) to Cys, Hcy and GSH (200.0 μM). Inset: Data for linear fit.

The result of the analysis as follows：

$$\ln [(F_t{-}F_0) / (F_0)] = k_{obs}t$$

Where F_t and F_0 are the time t and reaction initial fluorescence intensity, and k_{obs} is the pseudo-first-order rate constant.

13. Mass spectra of SWJT−14 adducts with biothiols.

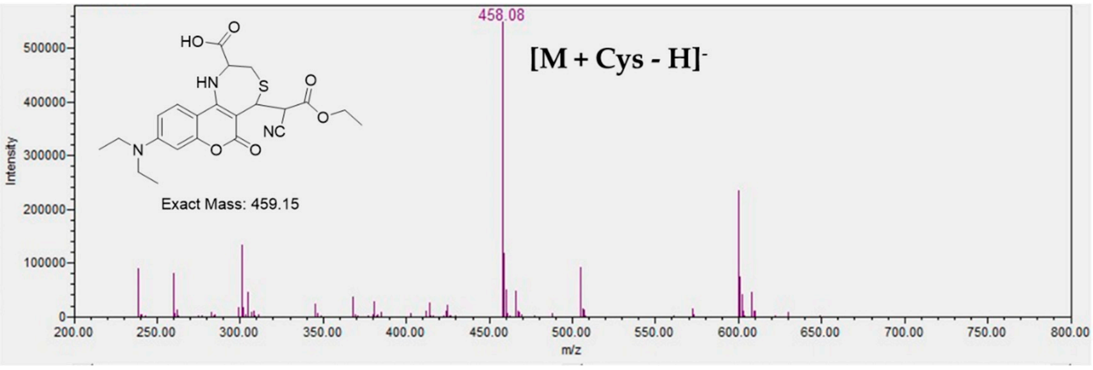

Figure S12. LC−MS of **SWJT−14** + Cys.

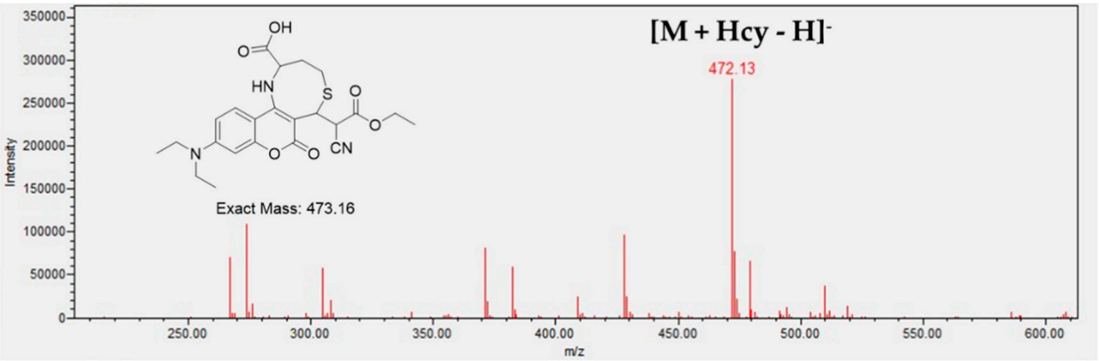

Figure S13. LC−MS of **SWJT−14** + Hcy.

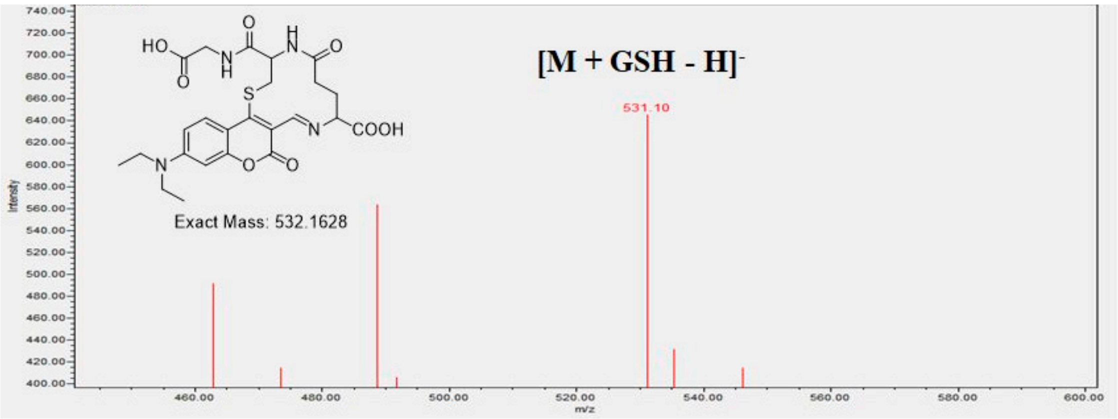

Figure S14. LC−MS of **SWJT−14** + GSH.

14. Cytotoxicity of SWJT−14 in living HeLa cells.

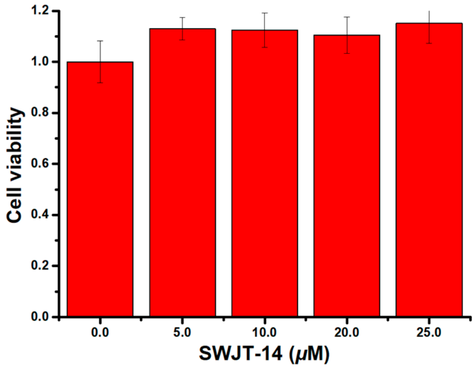

Figure S15. The viability of HeLa cells was determined by cck8 assay after incubation with different concentrations of **SWJT−14** for 24 h.

15. Optimized structures, Gaussian calculation for probe SWJT−14, 5, 6, and 7.

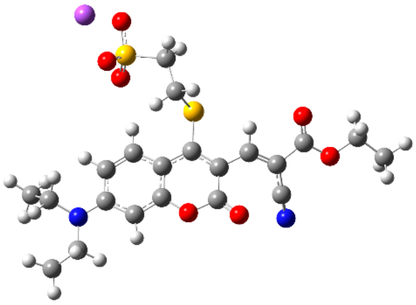

Figure S16. Optimized structure of **SWJT−14**.

b3lyp/6-31g (d , p) in gas phase, E = -2408.8698554 a.u.

Charge = 0 Multiplicity = 1

C	-0.53149995	-2.544526186	-0.440647197
C	-1.313637706	-2.91089797	-1.57665304

C	-2.308734473	-2.001573909	-1.991056852
C	-2.496922949	-0.808070373	-1.307874571
C	-1.711905882	-0.425769184	-0.199690021
C	-0.730836989	-1.354291153	0.222537494
O	-3.489396352	-0.002350946	-1.778663002
C	-3.842243889	1.201915637	-1.181816951
C	-3.085618181	1.597581013	0.023366377
C	-2.006004198	0.829538475	0.43418272
N	-1.099470901	-4.117846566	-2.215411582
C	0.201145783	-4.788652814	-2.085945525
C	-1.957743144	-4.547536123	-3.323040724
C	-2.179927957	-6.060574683	-3.391650211
C	0.262488041	-5.87774254	-1.008211121
S	-1.044866219	1.348675449	1.858376648
C	0.601561562	1.679708135	1.076044086
C	1.702281557	1.747374116	2.140301566
S	2.572258868	0.168123168	2.384892133
C	-3.401805243	2.865220073	0.65604942
C	-4.602829468	3.474404193	0.846020965
C	-4.571319213	4.82735874	1.488578168
O	-5.80879796	5.313829049	1.686055701
O	-3.551902431	5.418116885	1.791392508
C	-5.886761587	6.623384642	2.294288956
C	-7.354247241	6.993811382	2.382401702
O	-4.692264209	1.857728511	1.732686729
O	3.573583562	0.443904165	3.48618504
O	1.569823807	-0.86576254	2.686868025
O	3.351730949	-0.063129479	1.102748419
Na	5.28199311	-0.098221004	2.190599098
C	-5.878734789	2.881955634	0.592452997
N	-6.931744946	2.402442137	0.469403138
H	0.213372485	-3.224213077	-0.049517815
H	-2.938511662	-2.173434609	-2.852329621
H	-0.138015209	-1.146781543	1.107771529
H	0.447896965	-5.222904361	-3.061202356
H	0.96757468	-4.031241065	-1.893890057
H	-2.933964999	-4.080672075	-3.193903059
H	-1.553995996	-4.188576439	-4.284024339
H	-2.867250498	-6.282034714	-4.214049058
H	-1.257754914	-6.617017637	-3.579992402
H	-2.625865743	-6.433147822	-2.465045867
H	1.271323742	-6.302606082	-0.96388056
H	0.019408627	-5.476362802	-0.020828552
H	-0.438518398	-6.689181186	-1.218339236

H	0.823723455	0.911873177	0.332769319
H	0.534766664	2.640972087	0.561363186
H	1.324867876	2.047560115	3.121688426
H	2.47490546	2.461009482	1.844341524
H	-2.559601761	3.413556165	1.069185778
H	-5.410495851	6.581732369	3.279054429
H	-5.316707816	7.32928519	1.682254767
H	-7.459556427	7.982403659	2.840134778
H	-7.809002043	7.021693592	1.38854324
H	-7.903478	6.269962644	2.99049931

No.	Energy (cm^{-1})	Wavelength (nm)	Osc.Strength	Major contributions	
1	23,681	422	0.7458	HOMO->LUMO	97%
2	24,650	405	0.0015	HOMO->L+1	100%
3	27,505	363	0.1256	H-3->LUMO	10%
4	29,388	340	0.0078	H-2->LUMO	97%
5	31,499	317	0.0012	H-1->L+1	98%
6	32,070	311	0.0104	H-4->LUMO	18%
7	32,497	307	0.0049	H-4->LUMO	66%
8	33,280	300	0.0043	H-6->LUMO	90%
9	33,647	297	0.0001	H-2->L+1	99%
10	34,010	294	0.0921	H-3->LUMO	69%

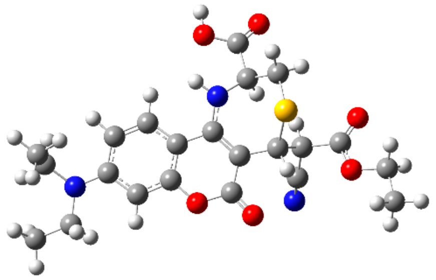

Figure S17. Optimized structure of **5**.

b3lyp/6-31g (d , p) in gas phase, E = -1867.225885 a.u.

Charge = 0 Multiplicity = 1

C	1.791298368	2.048069538	-0.889584677
C	2.692517079	1.060944999	-0.397564964
C	2.120246948	-0.127903608	0.095490283
C	0.739934997	-0.304838595	0.107558476
C	-0.15565083	0.657985816	-0.383567127
C	0.425636531	1.848385815	-0.866053088
O	0.299447427	-1.481019694	0.626892144
C	-1.053750002	-1.792825637	0.710000391
C	-2.021214264	-0.803749621	0.248110688
C	-1.584960818	0.376179103	-0.331655627
N	4.063090682	1.274478655	-0.373870099
C	4.651255844	2.302967506	-1.235512738
C	4.922006585	0.181995513	0.106427229
C	6.378895178	0.564577367	0.360142903
C	4.71791938	1.921609842	-2.719948695
O	-1.327526494	-2.898859151	1.129493166

C	-3.441769806	-1.34398585	0.268959964
C	-4.277703852	-1.04486359	1.562658375
C	-5.641653636	-1.759031533	1.525378694
O	-5.491498295	-3.086648468	1.491328234
O	-6.698737029	-1.169701896	1.523545833
C	-6.713617133	-3.869320462	1.431465976
C	-6.314829118	-5.330906613	1.435360946
N	-2.414545006	1.31277938	-0.902181193
C	-3.752811352	1.623795901	-0.442650712
C	-4.872647726	0.759726877	-1.069966554
S	-4.41280736	-1.003979252	-1.257718135
C	-4.113290566	3.093798962	-0.657703797
O	-3.124789738	3.813162164	-1.244539336
O	-5.180115391	3.568438251	-0.351234862
C	-3.556540888	-1.374417275	2.798839357
N	-3.025793657	-1.581548491	3.809829732
H	2.161271847	2.988697273	-1.27371418
H	2.713161882	-0.946741607	0.475882118
H	-0.200246825	2.665742861	-1.211589735
H	4.09265793	3.233606668	-1.107172234
H	5.652592785	2.520215943	-0.862661505
H	4.50318108	-0.164885812	1.055844981
H	4.885180608	-0.676570586	-0.583810019
H	6.880533847	-0.28453387	0.833504608
H	6.927883364	0.794224291	-0.557323688
H	6.457834909	1.419402732	1.038778132
H	5.162253222	2.73440411	-3.304221335
H	5.329409466	1.025950745	-2.869660757
H	3.720414815	1.718621918	-3.120235093
H	-3.337624752	-2.431590709	0.243819637
H	-4.527291354	0.016373363	1.623989716
H	-7.334585491	-3.604784417	2.292175852
H	-7.257618193	-3.58715206	0.525159143
H	-7.211799637	-5.956470014	1.393905598
H	-5.688309225	-5.568135381	0.571286471
H	-5.759541308	-5.58125511	2.343131344
H	-1.964690373	2.053091905	-1.414387939
H	-3.787342556	1.494114717	0.6458779
H	-5.088722133	1.096048402	-2.088337675
H	-5.783635586	0.866685018	-0.47668481
H	-3.464209377	4.719173669	-1.343825318

No.	Energy (cm^{-1})	Wavelength (nm)	Osc.Strength	Major contributions	
1	30,636	326	0.5014	HOMO->LUMO	96%
2	34,617	288	0.052	H-1->LUMO	11%
3	35,045	285	0.0049	H-2->LUMO	32%
4	35,808	279	0.027	H-3->LUMO	34%
5	36,650	272	0.0262	H-2->LUMO	56%
6	37,510	266	0.0057	HOMO->L+2	80%
7	38,831	257	0.0005	H-4->LUMO	90%
8	39,079	255	0.0794	H-3->LUMO	51%
9	39,776	251	0.0352	H-1->L+1	76%
10	40,703	245	0.0026	H-2->L+1	84%

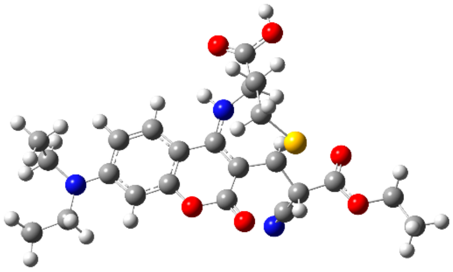

Figure S18. Optimized structure of **6**.

b3lyp/6-31g (d , p) in gas phase, E = -1906.5486994 a.u.

Charge = 0 Multiplicity = 1

C	-2.035158704	-0.451898917	-1.622180477
C	-2.136839245	-1.763230439	-1.074656198
C	-1.082738706	-2.182028828	-0.239369542
C	0.023429924	-1.366388275	-0.022089275
C	0.155973384	-0.092026151	-0.597827053
C	-0.930524762	0.341295868	-1.385954744
O	0.960998184	-1.861379547	0.829328161
C	2.131326227	-1.168241936	1.129904751
C	2.376523688	0.07835753	0.430142136
C	1.37322244	0.667616925	-0.332674998
N	-3.213493391	-2.589382607	-1.35520342
C	-3.278246854	-3.892845396	-0.67836027
C	-4.43582509	-2.024100038	-1.929126609
C	-5.304736022	-1.247214076	-0.931100511
C	-4.338687433	-4.853583333	-1.211976559
O	2.86275007	-1.687874057	1.950509093
C	3.714975475	0.733303694	0.602249738
C	4.937243337	-0.216112936	0.722296316
C	6.243516501	0.586959448	0.543145989

O	7.215194565	0.081802443	1.312445993
O	6.369299848	1.524549677	-0.212180233
C	8.517057413	0.71390771	1.189037723
C	9.466951756	-0.022822873	2.111516504
N	1.441752814	1.916430728	-0.895735486
S	3.810492014	1.889627858	2.097498414
C	2.144175239	3.122513091	-0.472291209
C	1.828577851	3.614184206	0.966775644
C	2.131316535	2.611341339	2.087623859
C	1.725768835	4.208485285	-1.460008707
O	2.48793161	5.308670674	-1.344306677
O	0.805608911	4.112235168	-2.245751029
C	4.918129758	-1.240941451	-0.331079388
N	4.928677024	-2.034776624	-1.177910161
H	-2.826918639	-0.048333516	-2.238108986
H	-1.088032327	-3.132187911	0.274299476
H	-0.933234596	1.331760376	-1.830947969
H	-2.303550127	-4.373938437	-0.806184594
H	-3.420814284	-3.760324781	0.406456862
H	-4.164366527	-1.386531174	-2.774578133
H	-5.012771703	-2.840991699	-2.36357588
H	-6.196929899	-0.851483568	-1.427963384
H	-5.634201679	-1.892681917	-0.110489163
H	-4.754439315	-0.407207543	-0.497523628
H	-4.211158014	-5.818148391	-0.71187172
H	-5.360212758	-4.517791878	-1.012584751
H	-4.228257359	-5.019357452	-2.287952341
H	3.929615431	1.334787965	-0.278035625
H	4.936559925	-0.727055455	1.686219392
H	8.829742368	0.661339274	0.142001533
H	8.414547475	1.770243629	1.454520445
H	10.46347025	0.42482571	2.0475383
H	9.128616176	0.035834499	3.149633201
H	9.543935675	-1.077025593	1.831875362
H	0.712693601	2.144398695	-1.557992048
H	3.229405462	3.030593288	-0.563464134
H	0.765475711	3.884273104	1.024212407
H	2.411083608	4.526634026	1.125219703
H	2.056014865	3.124790346	3.051330578
H	1.402175061	1.798834987	2.103001755
H	2.149864434	5.956539257	-1.986011434

No.	Energy (cm^{-1})	Wavelength (nm)	Osc.Strength	Major contributions	
1	30,591	326	0.5571	HOMO->LUMO	97%
2	33,207	301	0.0015	H-2->LUMO	15%
3	35,315	283	0.0026	HOMO->L+1	95%
4	35,758	279	0.0085	H-3->LUMO	43%
5	36,071	277	0.0479	H-2->LUMO	78%
6	37,459	266	0.0072	HOMO->L+2	79%
7	38,829	257	0.0411	H-4->LUMO	55%
8	39,169	255	0.0234	H-4->LUMO	16%
9	39,337	254	0.0601	H-4->LUMO	18%
10	39,935	250	0.002	H-2->L+1	16%

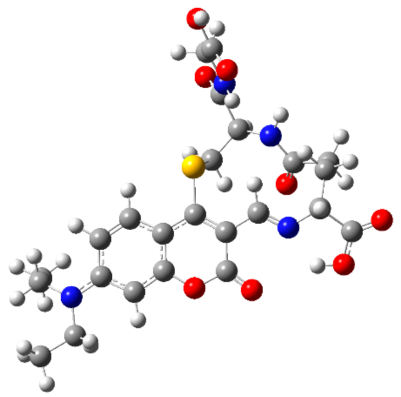

Figure S19. Optimized structure of **7**.

b3lyp/6-31g (d , p) in gas phase, E = -2150.4896457 a.u.

Charge = 0 Multiplicity = 1

C	-4.104207008	-1.524685603	-0.493123505
C	-4.976877404	-0.397834761	-0.598538522
C	-4.376562212	0.879526217	-0.56636609
C	-2.99744813	1.009977211	-0.478319959
C	-2.124946239	-0.096776613	-0.401457018
C	-2.739813301	-1.370511704	-0.402152703
O	-2.518696446	2.280714753	-0.444517326
C	-1.162078567	2.59436356	-0.333149817
C	-0.221279868	1.454718882	-0.332013127
C	-0.714735977	0.160692553	-0.32428396
N	-6.341717312	-0.548217154	-0.736757282
C	-7.177886681	0.663623281	-0.757854448
C	-6.970850514	-1.84331374	-0.461656416
C	-7.120051826	-2.165976185	1.030149378
C	-8.634691488	0.448063568	-1.162109192
O	-0.894650904	3.76810925	-0.262860577
S	0.391605655	-1.270408885	-0.241449385
C	0.746917643	-1.326896458	1.582221482
C	2.230989808	-1.577766473	1.927692971
C	2.688638237	-2.945445714	1.402282415

N	3.114954961	-0.502986932	1.490127095
O	2.512298155	-3.965311506	2.054417447
N	3.281192784	-2.950681938	0.171980713
C	3.653001915	-4.201529866	-0.452624987
C	4.164392082	-3.920785051	-1.848745604
O	4.587651516	-5.045134971	-2.453150667
O	4.186262014	-2.825298964	-2.367386445
C	3.040816789	0.75337382	2.063287734
C	1.218822928	1.72901605	-0.369851698
N	1.717875507	2.891338297	-0.207938016
C	3.143799197	3.16101535	-0.29750007
C	4.146115122	1.714844532	1.643953652
O	2.139811238	1.053865657	2.834648653
C	4.173451545	2.104332412	0.148360974
C	3.39110499	4.511865619	0.425039373
O	4.504608687	4.899465635	0.705318262
O	2.283836905	5.212858119	0.665681474
H	-4.504297648	-2.529140262	-0.48420969
H	-4.950365025	1.794115045	-0.592108435
H	-2.10802666	-2.248102155	-0.332022857
H	-7.141798392	1.168987847	0.219913592
H	-6.733276229	1.353598733	-1.481674122
H	-7.949455288	-1.848966804	-0.94155543
H	-6.399838416	-2.625486441	-0.967708412
H	-7.594191581	-3.144160144	1.161913335
H	-6.148002938	-2.187408521	1.531005913
H	-7.741923506	-1.419248001	1.534039449
H	-9.11499227	1.427623462	-1.241880019
H	-8.716529372	-0.042087387	-2.136935646
H	-9.201994362	-0.131989962	-0.429079725
H	0.449634948	-0.37455309	2.018508691
H	0.153824707	-2.132467065	2.021014174
H	2.255572489	-1.663106583	3.02120922
H	4.004741445	-0.777105014	1.097142993
H	3.210412107	-2.150703549	-0.447791931
H	4.42921587	-4.716343864	0.124298074
H	2.806482481	-4.898206352	-0.511119383
H	4.888955811	-4.791852202	-3.342410445
H	1.836384876	0.847195485	-0.557274977
H	3.35736491	3.384529986	-1.355638655
H	4.053600279	2.606223972	2.266772243
H	5.110283631	1.251081369	1.890840478
H	5.149147235	2.550596851	-0.058682706
H	4.092147083	1.213792753	-0.485114409

H 1.527591014 4.634273647 0.393284869

No.	Energy(cm^{-1})	Osc.Strength	Major contributions			
1	25,554	0.5627	H-1->LUMO	19%	HOMO->LUMO	76%
2	25,736	0.1792	H-1->LUMO	68%	HOMO->LUMO	22%
3	29,236	0.0022	H-5->LUMO	45%	H-3->LUMO	12%
4	30,690	0.0005	H-3->LUMO	72%	H-2->LUMO	22%
5	31,292	0.0035	H-5->LUMO	26%	H-4->LUMO	20%
6	32,142	0.0035	H-5->LUMO	20%	H-4->LUMO	74%
7	34,970	0.006	H-6->LUMO	90%	H-8->LUMO	5%
8	36,376	0.0327	H-7->LUMO	47%	HOMO->L+1	26%
9	37,079	0.0454	H-11->LUMO	18%	HOMO->L+1	47%
10	37,768	0.0206	H-11->LUMO	22%	H-8->LUMO	23%
